# Photosynthetic response and antioxidative activity of ‘Hass’ avocado cultivar treated with short-term low temperature

**DOI:** 10.1038/s41598-022-15821-3

**Published:** 2022-07-08

**Authors:** Sun Woo Chung, Hyungmin Rho, Chan Kyu Lim, Mi Kyoung Jeon, Seolah Kim, Yeon Jin Jang, Hyun Joo An

**Affiliations:** 1 Research Institute of Climate Change and Agriculture, National Institute of Horticultural and Herbal Science, Jeju, 63240 Republic of Korea; 2 Department of Horticulture and Landscape Architecture, National Taiwan University, Taipei, 10617 Taiwan

**Keywords:** C3 photosynthesis, Abiotic

## Abstract

To investigate the effects of short-term low temperatures, three-year-old avocado (*Persea americana* cv. Hass) seedlings were treated with 1, − 2, or − 5 °C for 1 h and subsequently recovered in ambient condition for 24 h. Leaf color changes were investigated with chlorophyll, carotenoid, and phenolic contents. Photosynthetic responses were examined using gas exchange analysis. With H_2_O_2_ contents as oxidative stresses, enzymatic (ascorbate peroxidase, APX; glutathione reductase, GR; catalase, CAT; peroxidase, POD) and non-enzymatic antioxidant activities were determined using spectrophotometry. Leaves in the avocado seedlings started to be discolored with changes in the contents of chlorophyll *a*, carotenoids, and phenolics when treated with − 5 °C. However, the H_2_O_2_ content was not different in leaves treated with low temperatures. Photosynthetic activities decreased in leaves in the seedlings treated with − 5 °C. Of antioxidant enzymes, APX and GR have high activities in leaves in the seedlings treated with 1 and − 2 °C. In leaves in the seedlings treated with − 5 °C, the activities of all enzymes decreased. Non-enzymatic antioxidant activity was not different among leaves treated with low temperatures. These results indicated that APX and GR would play a critical role in withstanding chilling stress in ‘Hass’ avocado seedlings. However, under lethal temperature, even for a short time, the plants suffered irreversible damage with the breakdown of photosystem and antioxidant system.

## Introduction

Avocado (*Persea americana* Mill) is one of the most important cash crops in international trade and contributes substantially to the livelihoods of smallholders in many countries. In 2020, avocado was cultivated on the land of 807,469 hectares (ha) worldwide, and the global production reached 8.06 million (m) tonnes (t) in 2020, about a two-fold increase from a decade prior^1^. The top five producing countries were: Mexico with 2.39 mt, Colombia with 0.88 mt, Dominican Republic with 0.67 mt, Peru with 0.66 mt, and Kenya with 0.32 mt^1^. The yields varied depending on the producing countries, from 33.57 t ha^−1^ in El Salvador to 2.45 t ha^−1^ in Dominica^1^. In addition to the traditional cultivation area, other countries are trying to cultivate avocado due to the increase in domestic consumer interest in the fruit with health benefits and high valuable nutrients.

Avocado belongs to the Lauraceae family and originated in subtropical/tropical regions, including Costa Rica, Guatemala, and Mexico. The horticultural varieties of avocado are well differentiated based on morphology and their climatic adaptation: *P*. *americana* var. drymifolia (Mexican), native to subtropical regions, is the most low-temperature tolerant, *P*. *americana* var. guatemalensis (Guatemalan) is intermediate, and *P*. *americana* var. americana (West Indian), originating from tropical regions, is the least low-temperature tolerant^2,3^. Mexican and Mexican × Guatemalan hybrid^4^, including 'Fuerte', ‘Hass’, and their genetic variants, are predominant commodities in the avocado industry, which have been moved to and well adapted in Mediterranean regions, including California in the United States, Israel, South Africa, Turkey, and Chile^5^. Of these varieties, ‘Hass’ cultivar is the most commercially popular and comes from a graft, a mixture of an advanced hybrid of the Guatemalan and Mexican varieties, allowing it to thrive in temperate climates^6^. However, the avocado production system does not currently have a validated technological model, including pre- and post-harvest for new cultivation area, which would affect yield and fruit quality^7^.

Avocado has a lifespan of decades, and therefore long-term agricultural planning is critical, especially considering the anticipated effects of climate change. The Intergovernmental Panel on Climate Change Global estimates global warming of 1.2 up to 3.0 °C by 2050, depending on greenhouse gas emission pathways^8^. Such changes in temperature climates would cause shifts in the cultivation regions with new agricultural practices since high temperatures could restrict the growth of commercial avocado cultivars, including ‘Hass’ and ‘Fuerte’^9^. In temperate countries, including Korea and Japan, avocado cultivation becomes possible by reaching the climatic requirements due to the increase in average ambient temperatures. The cultivation range of ‘Hass’ cultivar would also be expected to expand temperate regions of America continent^10^. However, lower temperatures in the areas than their origins would not be suitable for avocado to overwinter. Furthermore, the changing climate also brings about frequent chilling events in temperate regions^11^, poses a question in adapting avocado trees to the region. Recently, in temperate regions, greenhouse cultivation is essential and prevalent for protecting avocado from low temperatures of winter or early spring seasons; however, there is no information on proper temperature ranges considering heating costs. In particular, at the early growing stages, avocado is more vulnerable to chilling- or freezing-low temperatures; thus, a better understanding of young avocado under low temperatures is required to provide necessary information for sustainable avocado cultivation in the new cultivation regions.

Low temperature causes chilling stress, resulting in significant yield reduction and crop losses^12^. Chilling stress diminishes the rate and efficiency of photosynthesis, caused by a decrease in carbon dioxide (CO_2_) diffusion from the atmosphere to the site of carboxylation^13^. In addition to the CO_2_ diffusion rate, photosynthesis also is lowered due to a decrease in ribulose-1,5-bisphosphate carboxylase/oxygenase (RuBisCo) activity and other metabolic changes, limiting carboxylation efficiency^14^. The limitation derived from these metabolic constraints is non-stomatal limitation, or biochemical limitation, while that derived from CO_2_ diffusion is stomatal limitation. In addition, leaf CO_2_ diffusion capacity decreases in response to a sequence of resistance while diffusing from the intercellular cavities to the site of carboxylation in the chloroplast, which is referred to as mesophyll limitation^15^. These three factors can co-limit the rate of photosynthesis, while the proportion of the three limitations can vary on the intensity or duration of chilling stress.

Under low temperatures, biochemical and biophysical limitation factors become the primary constraint, with which the reduced photosynthetic activities cannot process the light energy when treated with high light intensity during the day, although several protection mechanisms, e.g., dissipation by heat, chlorophyll fluorescence, etc., accompany photoinhibition to deal with excess light energy. When low temperatures are combined with or followed by exposure to light, a decrease in CO_2_ diffusion causes the over-excitation of the reaction centers of the photosystems and the formation of reactive oxygen species (ROS)^3^. ROS include a free radical, such as hydrogen peroxide (H_2_O_2_), hydroxyl radical (OH^−·^), singlet oxygen (^1^O_2_), and superoxide (O_2_^−·^), and act as damaging, protective, or signaling factors depending on the sophisticated equilibrium between ROS production and scavenging at the proper site and time^16^. Chilling stress induces excessive ROS formation leading to the disruption of cell organelles and ultimately cell death^16,17^. Antioxidant enzymes for ROS scavenging and the synthesis of these enzymes are enhanced while oxidative stresses are generated^18^. In enzymatic antioxidant system, ascorbate peroxidase (APX; EC 1.11.1.11) employs ascorbate as the specific electron donor to scavenge H_2_O_2_ to H_2_O and glutathione peroxidase (GPX; EC 1.11.1.9) catalyzes the reduction of H_2_O_2_ and HO_2_ to H_2_O and fatty alcohols, respectively, using thioredoxin as an electron donor. Glutathione reductase (GR; EC 1.6.4.2) catalyzes the reduction of oxidized glutathione (GSSG) to reduced glutathione (GSH). Superoxide dismutase (SOD; EC 1.15.1.1) catalyzes the elimination of O_2_^−·^ by reducing it into O_2_ and H_2_O_2_, catalase (CAT; EC 1.11.1.6) switches the H_2_O_2_ into H_2_O and O_2_, and peroxidase (POD; EC 1.11.1.7) works in the extra-cellular pace for scavenging H_2_O_2_^17^. In addition to enzymatic antioxidants, non-enzymatic antioxidants, including phenolics, carotenoids, tocopherol, and proline, can also counteract stress-induced ROS^17^. Of these, carotenoids and phenolics are widely distributed and structurally diverse metabolites involved in many stress responses as well as pigmentation^19^. Carotenoids have been associated with the protection of chlorophyll from oxidation against ROS in the photosystems and the light harvesting complexes^20^. Phenolics have ideal structures for scavenging ROS, and they have been shown to be more effective antioxidants than tocopherols and ascorbates on in vitro experiments^21^. It can also form complexes with metals and raise the activity of antioxidative enzymes to scavenge ROS^22^.

Thus, we investigated low threshold temperatures that may physiologically induce chilling stress to avocado seedlings. Photosynthetic responses were characterized in ‘Hass’ avocado seedlings after exposure to short-term low temperatures. We also determined oxidative stress and antioxidant activities under the same condition. Through these photosynthetic and antioxidant analyses, we intended to characterize the development of chilling stress over different low temperatures and subsequently identify the critical chilling temperature at the early growing stage in avocado trees.

## Materials and methods

### Plant materials and experimental designs

In December 2019, ‘Hass’ avocado seeds were sowed in cylindrical black plastic pots (200 mm in diameter, 300 mm in length, and 30 L in volume) in medium containing 90% commercial grow media (4% zeolite, 7% perlite, 6% vermiculite, 68% coco peat, and 14% peat moss) (Baroker, Seoul Bio Co., Ltd., Gyeongju, Republic of Korea) and 10% coarse sand (by volume), and were grown in a greenhouse at the experimental orchard of the Research Institute of Climate Change and Agriculture, the National Institute of Horticultural and Herbal Science, Jeju (33° 28′ N, 126° 31′ E), Republic of Korea. After seeds sprouted, the seedlings were irrigated weekly by hand drip and fertigated bi-monthly with Yara Milla (12% N, 11% P_2_O_5_, 18% K_2_O, 2.65% MgO, 19.9% SO_3_, 0.02% Zn, and 0.015% B) (Yara, Oslo, Norway). Crop management practices were performed according to the guideline^23^ of the Rural Development Administration, Republic of Korea. We chose uniform seedlings of BBCH (Biologische Bundesanstalt, Bundessortenamt und Chemische Industrie) code 115^24^ for examining photosynthetic responses and antioxidant activities, compared before and after low-temperature treatments. Since the photosynthetic responses and antioxidant activities were measured using non-destructive and destructive methods, respectively, different avocado seedlings were used for each analysis. Before low-temperature treatment, six and three avocado seedlings were examined for photosynthetic responses and antioxidant activities, respectively, on February 20, 2021. Three biological replicates of each analysis were treated with low temperatures, including 1, − 2, or − 5 °C, for 1 h in dark chambers on March 15, 2021. Then all the plants were relocated to the greenhouse to recover from the chilling stress for 24 h before investigating photosynthetic responses and antioxidant activities. Photosynthetic responses were measured with a recently fully expanded leaf on each biological replicate. Antioxidant activities were biochemically assayed with five leaves of each replicate. These leaves were frozen in liquid nitrogen at stored at − 80 °C until use.

**Figure 1:**
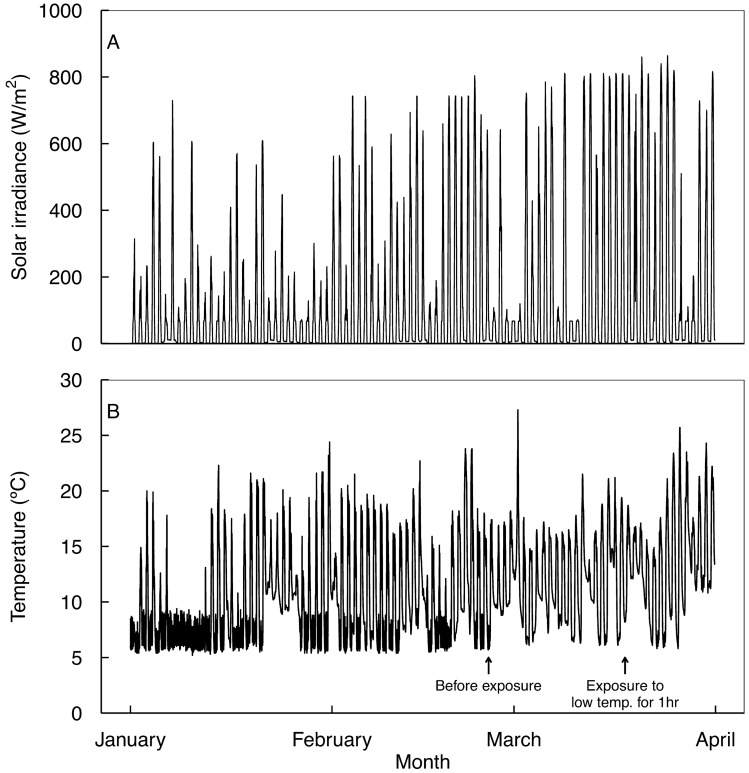
Solar irradiance (**A**) and temperature (**B**) from 01 January to 31 March 2021 in a greenhouse at the experimental orchard of the Research Institute of Climate Change and Agriculture, the National Institute of Horticultural and Herbal Science, Jeju (33° 28′ N, 126° 31′ E), Republic of Korea. ‘Hass’ three-year-old avocado seedling before treatment and treated to low temperatures were cultivated with the average growing temperature of 10.53 °C and 11.06 °C, respectively.

Daily mean solar irradiance and temperature in the greenhouse were measured and recorded from January 01 to March 31, 2021 (Fig. 1) using data loggers (WatchDog 2450, Spectrum Technologies, Inc., Aurora, IL, USA) with photosynthetically active radiation sensors (LightScout Quantum Light Sensor, Spectrum Technologies, Inc.).

### Determination of total chlorophyll, carotenoid, and phenolic contents

Total chlorophyll and carotenoid contents were determined following the procedures of Lichtenthaler^25^. The absorbance of the extracts was measured at wavelengths of 661.1, 644.8, and 470 nm using a spectrophotometer (OPTIZEN™ Alpha, K Lab Co., Ltd., Daejeon, Republic of Korea). Chlorophyll and carotenoid contents were calculated based on µg g^−1^ fresh weight (FW) using Eqs. (1), (2), (3), and (4).

1 $$Chlorophyll \, a = 11.24 \, A_{\text{661.6}} - 2.05 \, A_{\text{644.8}},$$

2 $$Chlorophyll \, b = 20.13 \, A_{\text{644.8}} - 2.05 \, A_{\text{661.6}},$$

3 $$Total \, chlorophylls = 7.05 \, A_{\text{661.6}} + 18.09 \, A_{\text{644.8}},$$

4 $$Total \, carotenoids = \frac{\left(\mathrm{1000 } \, A_{470} - 1.90\, chlorophyll \, a - 63.14\, chlorophyll \, b\right)}{214}.$$

Total phenolic contents were measured using Folin-Ciocalteu assay as described by George et al.^26^. To each extract concentration of leaf extracts (25, 50, 100, 200, and 400 µg), 2.5 mL of Folin-Ciocalteu reagent (1/10 dilution) and 2 mL of 7.5% Na_2_CO_3_ (w/v) solution were added and incubated at 45 °C for 15 min and the absorbance was read at a wavelength of 765 nm using a spectrophotometer (OPTIZEN™ Alpha, K Lab Co., Ltd., Daejon, Republic of Korea). The total phenolic content was expressed as mg gallic acid equivalents per gram of FW (mg GAE g^−1^ FW).

### Gas exchange and chlorophyll fluorescence measurements

All gas exchange and chlorophyll fluorescence measurements were simultaneously conducted for recently fully expanded leaves using a portable photosynthesis system (LI-6400XT, Li-Cor, Co., Inc., Lincoln, NE, USA) equipped with a leaf chamber fluorometer (LI-6400-40), according to Rho, et al.^15^. CO_2_ and light response curves (i.e., the net CO_2_ assimilation (*A)*—the intercellular CO_2_ concentration (*C*_i_) and *A*—photosynthetic photon flux density (*PPFD*) curves) of the leaves were determined before and after the low-temperature treatments. During the measurements, the leaf chamber was maintained at 20 °C, and the relative humidity of the leaf chamber was at around 50%. We recorded *A*, transpiration rate (*E*), *C*_i_, stomatal conductance to H_2_O (*g*_s_), the quantum yield of PSII estimated by fluorescence measurement (*Φ*_PSII_), the quantum yield estimated by gas exchange measurement (*Φ*_CO2_), photochemical quenching (qP), and non-photochemical quenching (qN) over these measurements. The nomenclatures for chlorophyll fluorescence parameters were referred to Baker^27^.

CO_2_ response curves were constructed as a function of *C*_i_ ranging from 0 to 1400 µmol CO_2_ mol^−1^. Under saturating light conditions of 1500 *PPFD*, the CO_2_ concentration of the leaf chamber head was first set to 400 µmol CO_2_ mol^−1^ (equivalent to ppm) and reduce to 200, 100, and 0 µmol CO_2_ mol^−1^ to measure the photosynthetic responses at low CO_2_ levels. Then, the *C*_a_ was set back to 400 µmol CO_2_ mol^−1^ and remained for 4 min to reacclimate RuBisCo to the ambient level of CO_2_. At 200 ppm intervals, the CO_2_ level was increased to 600, 800, 1000, 1200, and 1400 µmol CO_2_ mol^−1^ while the instrument’s collecting the parameters. Light response curves were constructed as a function of incident *PPFD* ranging from 50 to 1500 µmol m^−2^ s^−1^. Each *PPFD* was projected from internal red and blue light-emitting diodes with 10% blue light to maximize stomatal aperture and was maintained for 10 min for equilibration. The ambient CO_2_ concentration of 400 µmol CO_2_ mol^−1^ was used over the measurements. The light intensity of the leaf chamber was set to 0 µmol quanta m^−2^ s^−1^ *PPFD* to adapt the leaves in the dark for 20 min and then with the saturating pulse of 8000 µmol quanta m^−2^ s^−1^ *PPFD* for 0.5 s Fv/Fm was measured. The light intensity then was maintained at 1500 µmol quanta m^−2^ s^−1^ *PPFD* for 20 min to adapt the leaves in light to measure the light saturating photosynthesis and chlorophyll fluorescence values. The light intensity was decreased to 1200, 1000, 800, 600, 400, 200, 100, and 50 µmol quanta m^−2^ s^−1^ while the instrument’s collecting the parameters.

### Determination of H_2_O_2_ contents

H_2_O_2_ contents were determined as described by Shi et al.^28^. Leaves were homogenized in an ice bath with 0.1% trichloroacetic acid (TCA) using a tissue to TCA ratio at 1:10 (w/v). The homogenate was centrifuged at 12000×*g* for 15 min, and 0.5 mL supernatant was added to 0.5 mL of 10 mM potassium phosphate (pH 7.0) and 1 mL of 1 M KI. After 1 h in darkness, the absorbance was read at a wavelength of 390 nm, and the H_2_O_2_ contents were calculated based on the standard curve.

### Antioxidant enzyme extraction and assay

Leaves were ground with a mortar and a pestle in 1:15 (w/v) tissue to extraction buffer of 100 mM potassium phosphate (pH 7.5) containing 2 mM EDTA–Na_2_, 1% polyvinylpolypyrrolidone, 1 mM phenylmethylsulfonyl fluoride according to Anderson et al.^29^. Ascorbate (5 mM) and dithiothreitol (10 mM) were added for ascorbate peroxidase (APX) and glutathione reductase (GR) assay, respectively. The extracts were filtered through 0.45 µm polyethersulfone membranes (Millex Syringe Filter, MilliporeSigma, Burlington, MA, USA). The filtered homogenates were centrifuged at 15,000×*g* at 4 °C for 20 min. The supernatants were saved as crude enzyme extracts.

Protein content was determined with bovine serum albumin as a standard, according to Bradfor ^30^. Protein content and enzyme activities were measured by using a spectrophotometer (OPTIZEN™ Alpha).

APX activity was measured as described by Nakano and Asada^31^ using a reaction mixture containing 50 mM potassium phosphate (pH 7.0), 0.2 mM H_2_O_2_, and 0.5 mM ascorbate. The H_2_O_2_-dependent oxidation of ascorbate was monitored following the absorbance decrease at a wavelength of 290 nm. The enzyme activity was calculated by using the extinction coefficient of 2.8 mM^−1^ cm^−1^.

CAT activity was measured according to Mishra et al.^32^. A reaction mixture composed of 50 mM potassium phosphate (pH 7.0) and 11 mM H_2_O_2_. The decomposition of H_2_O_2_ was measured at a wavelength of 240 nm, and the enzyme activity was calculated by using the extinction coefficient of 0.036 mM^−1^ cm^−1^.

POD activity was determined by measuring the absorbance increase at a wavelength of 470 nm due to the formation of tetraguaiacol in a reaction mixture containing 40 mM potassium phosphate (pH 7.0), 1.5 mM guaiacol, and 6.5 mM H_2_O_2_ as described by Chance and Maehly^33^. The enzyme activity was calculated by using the extinction coefficient of 26.6 mM^−1^ cm^−1^.

GR activity was measured by oxidized GSH-dependent oxidation of NADPH. The reaction mixture contained 100 mM potassium phosphate (pH 7.8), 2 mM EDTA, 0.2 mM NADPH, and 0.5 mM GSSG^34^. The absorbance change at a wavelength of 340 nm was monitored and the enzyme activity was calculated by using the extinction coefficient of 6.22 mM^−1^ cm^−1^.

### Determination of non-enzymatic antioxidant activity

Non-enzymatic antioxidant activity was determined using ABTS radical scavenging assay, according to Re et al.^35^. ABTS was dissolved in distilled water to a 7 mM concentration. ABTS radical cation was produced by reacting the ABTS stock solution with 2.45 mM potassium persulfate and allowing the mixture to stand in the dark at room temperature for 12 h. The stock solution (25 µL) was diluted with 70% methanol (22 mL). The sample (50 µL) was mixed with the ABTS solution (1 mL) for 3 min. The absorbance was read at a wavelength of 734 nm. *A*_c_ is the absorbance of the control, reacted with ethanol (50 µL) and the ABTS solution (1 mL). *A*_s_ is the absorbance of the sample. The ABTS radical scavenging capacity was calculated using Eq. (5).

5 $$ABTS \, radical \, scavenging \, activity = 1 - \left[\frac{(Ac - As)}{Ac}\right]$$

### Statistical analysis

All statistical analyses were performed using R Statistical Software version 4.1.3^36^ and RStudio IDE version 2022.02.1 + 461^37^ with agricolae packages version 1.3–5^38^. Statistically significant differences were determined by analysis of variance. Means were compared using the Tukey’s honestly significant difference test at *p* < 0.05. Linear correlations between selected variables were determined by Pearson correlation coefficient (r), after Shapiro-Wilks test for validating the normal distribution of the variables. The difference was considered significant at *p* < 0.05.

## Results and discussion

### Visual appearances and pigment changes

**Figure 2:**
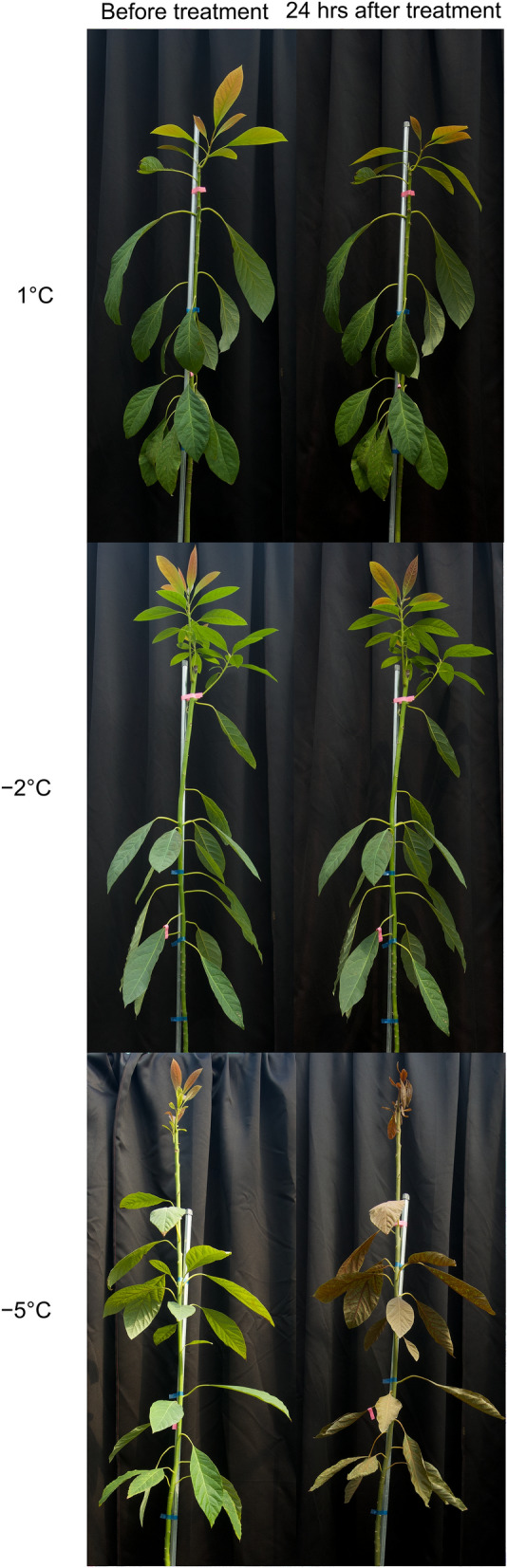
Three-year-old ‘Hass’ avocado seedlings before treatment (left) and 24 h after (right) treated with 1 (top), − 2 (middle), and − 5 °C (bottom) for 1 h.

‘Hass’ avocado seedlings were visually changed before and after treatment with 1, – 2, or – 5 °C (Fig. 2). The color of leaves in the seedlings treated with 1 and – 2 °C maintained green as same as leaves before treatment, while those treated with – 5 °C turned yellow or brown. Since avocado is an evergreen fruit tree, the color changes would be a visual signal for physiological disorders and leaf senescence^39^. The discoloration of leaves in the seedlings treated with – 5 °C for 1 h was shown in various avocado cultivars, including ‘Geada’, ’Fortuna’, ’Fuerte’, ’Quintal’, ’Margarida’ and ’Primavera’^40^. In grafted one-year ‘Hass’ avocado, exposure from – 2 to – 3 °C for 10 h led to the discoloration and death of leaves within one to three days^3^. It can also accompany the degradation of pigment, protein, and cell wall components, and a decrease in photosynthesis^39^. Although the visual damage showed a part of plant status since morphological changes would not fully describe the degree of damage, biochemical and physiological investigation are required to identify tree conditions exposed to various low temperatures.

The pigment compositions of leaves changed under low temperatures in various plants^20^. In the present study, we determined the changes in the concentrations of chlorophyll *a* and *b*, carotenoids, and phenolics (Table 1). The contents of chlorophyll *a* in the leaves in the seedlings treated with 1 and – 2 °C did not change compared to the leaves before treatment, while those treated with – 5 °C significantly decreased. The contents of chlorophyll *b* and total chlorophyll did not change between those before and after treatments (Table 1). Compared to leaves before treatment, the ratio of chlorophyll *a* to *b* decreased up to about 50% in the leaves in the seedlings treated with – 5 °C, but those treated with 1 and – 2 °C were not significantly different. Chlorophyll *a* dominates in photosystems, while chlorophyll *b* content is restricted to the light harvesting complex^20^. Therefore, a decrease in the chlorophyll *a* in the leaves in the seedlings treated with – 5 °C indicated that at a specific low temperature, even for a short time, whole photosystems were damaged rather than specific sites such as the light harvesting complex.

**Table 1:** Changes in chlorophyll, carotenoid, and phenolic contents of three-year-old ‘Hass’ avocado seedling leaves before treatment and 24 h after treated with 1, − 2, and − 5 °C for 1 h.

Treatment	Chlorophyll *a*	Chlorophyll *b*	Total chlorophyll	Ratio of Chlorophyll *a* to *b*	Total Carotenoid	Total phenolic
	µg g^–1^ FW				µg g^–1^ FW	mg GAE eq. g^–1^ FW
Before	129.87 ± 14.80^1^a^2^	98.19 ± 18.37a	228.06 ± 31.27a	1.38 ± 0.21a^3^	102.53 ± 20.56b	41.67 ± 4.66b
1 °C	132.37 ± 3.25a	94.51 ± 5.88a	233.41 ± 22.41a	1.41 ± 0.17a	102.43 ± 19.27b	44.34 ± 3.45b
− 2 °C	92.70 ± 14.77ab	82.27 ± 7.22a	174.98 ± 8.14a	1.14 ± 0.10ab	79.07 ± 8.14b	45.56 ± 3.45b
− 5 °C	70.55 ± 14.80b	107.15 ± 2.93a	177.70 ± 4.07a	0.65 ± 0.01b	260.98 ± 12.93 a	68.48 ± 5.04a
ANOVA	8.006 (0.008)***	0.981 (0.449)	2.539 (0.130)	5.728 (0.022)*	29.12 (0.000)***	8.677 (0.006)**

^1^Means with standard errors from three replicates with five leaf each.

^2^Means within columns followed by different lowercase letters are significantly different according to Tukey’s honestly significant difference test at *p* < 0.05.

^3^Statistical significance codes: *, **, and *** for *p* < 0.05, 0.001, and 0.001 levels, significantly.

When plants were exposed to low temperatures, secondary metabolites were synthesized to protect plant cells from ROS, which can cause changes in leaf color^20^. Total carotenoids and phenolics in the leaves in the seedlings treated with – 5 °C were accumulated more than the others (Table 1). Carotenoids and phenolics have been associated with the photoprotection against chlorophyll oxidation by ROS in the photosystems and light harvesting complexes^20^. Especially, phenolic compounds have dual roles as antioxidants and as substrates for oxidative browning reactions with oxidoreductases, including polyphenol oxidase (PPO) and POD^41^. They are functional as antioxidants at relatively low concentrations while, at higher concentrations, since they themselves are susceptible to oxidation, they can behave as pro-oxidants due to their involvement in initiation reactions. The main oxidative phenomenon (usually deteriorative) of this type is enzymatic browning which involves initial enzymatic oxidation of phenolic compounds located predominantly in the vacuole by PPO located in cytoplasm to form slightly colored quinones. Although the oxidation of phenolic compounds leads to yellowing or browning, the products are considered antioxidants. Therefore, the accumulation of carotenoids and phenolics in the leaves in the seedlings treated with – 5 °C were associated with severe damages compared to those treated with 1 and – 2 °C.

### Oxidative stress

**Figure 3:**
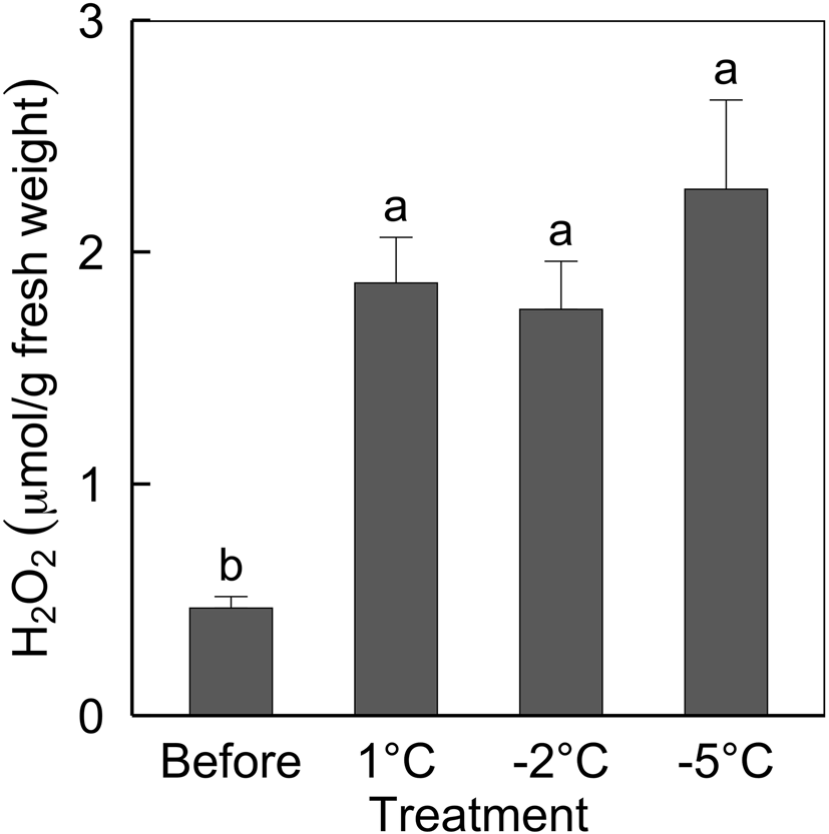
Hydrogen peroxide (H_2_O_2_) contents in the leaves of three-year-old ‘Hass’ avocado seedling before treatment and 24 h after treated with 1, − 2, and − 5 °C for 1 h. Means with standard errors from three replicates with five leaf each. Means with bars followed by different lowercase letters are significantly different according to Tukey’s honestly significant difference test at *p* < 0.05.

H_2_O_2_ contents, the index of oxidative stress, increased in the leaves in avocado seedlings treated with low temperatures (Fig. 3). The H_2_O_2_ contents in all the leaves were about four-fold higher than before treatment; however, the contents were not significantly different among the leaves in avocado seedlings treated with low temperatures. Enhanced ROS generation is a well-known effect of cold stress by low temperature^42^. When the ambient temperature becomes lower, the light becomes excessive due to the reduction of the photosynthetic activities, so there is excessive production of excited chlorophyll, which generates ROS. However, the effect of increased ROS generation on plant physiology varies from plant species, the level and duration of low temperatures, and other growing conditions. In this study, only avocado seedlings treated with – 5 °C did not survive (Fig. 1), despite the same levels of H_2_O_2_ contents among leaves in avocado seedlings treated with different low temperatures; the H_2_O_2_ contents were also not consistent with pigment contents (chlorophyll *a*, r = – 0.74^non-significant (ns)^; chlorophyll *b*, r = – 0.47^ns^; total chlorophylls, r = – 0.88^ns^; total carotenoids, r = 0.53^ns^; total phenolics, r = 0.67^ns^.). These results indicated that avocado seedlings would have different physiological or biochemical responses against oxidative stress at each low temperature.

### Leaf gas exchange properties

**Figure 4:**
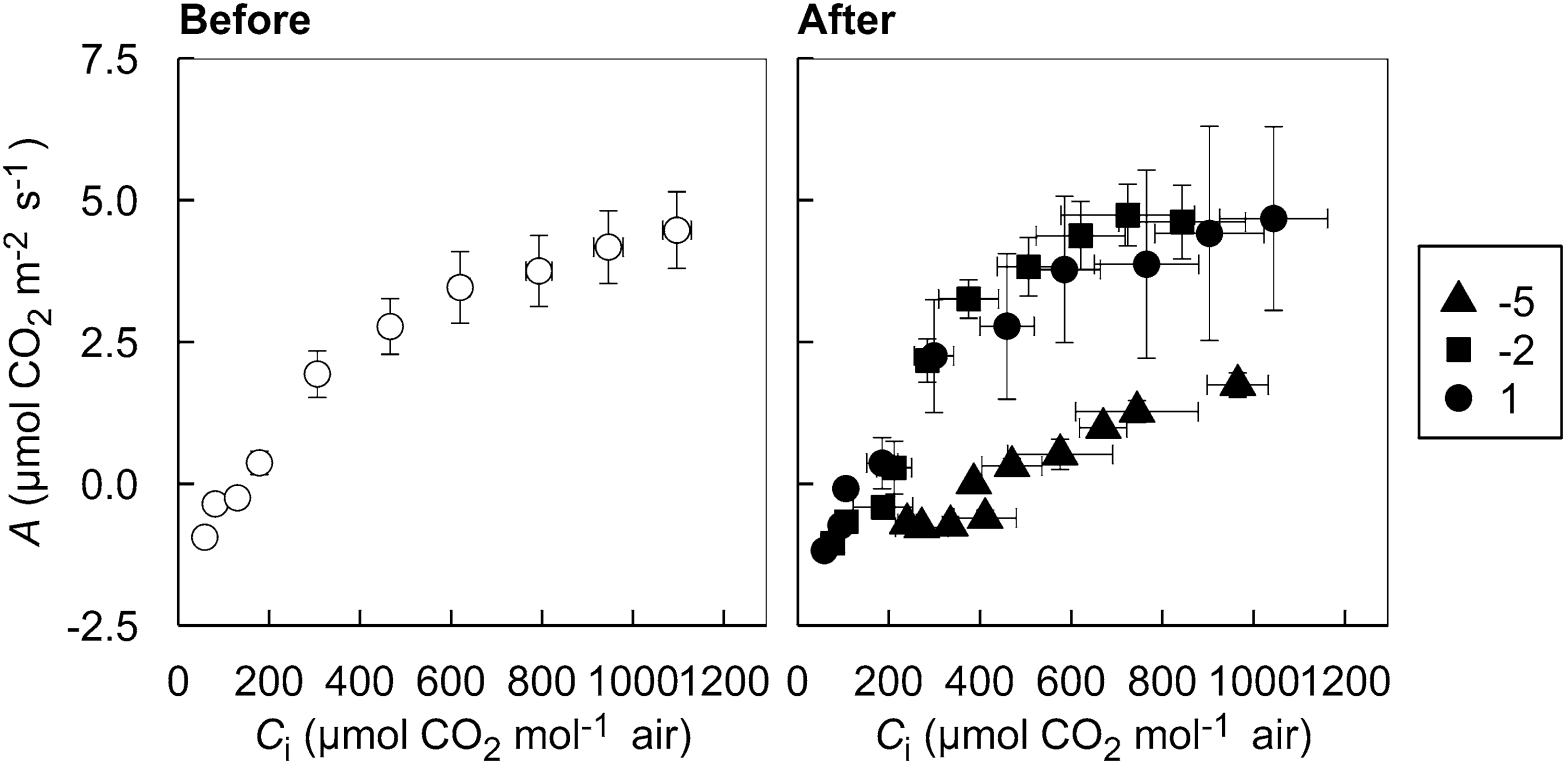
*A*–*C*_i_ curves, light-saturated net photosynthetic CO_2_ assimilation rate (*A*) as a function of intercellular CO_2_ concentration (*C*_i_) of three-year-old ‘Hass’ avocado seedling leaves before (left) and after (right) short-term low temperature treatments. All symbols present the mean responses of biological replications (*n* = 6 and 3 for before and after treatments, respectively). Error bars show the standard errors of the means for *A* (vertical) and *C*_i_ (horizontal). Closed triangles, squares, and circles indicate the responses of the samples after treated with 1 h 1, − 2, and − 5 °C, respectively.

**Figure 5:**
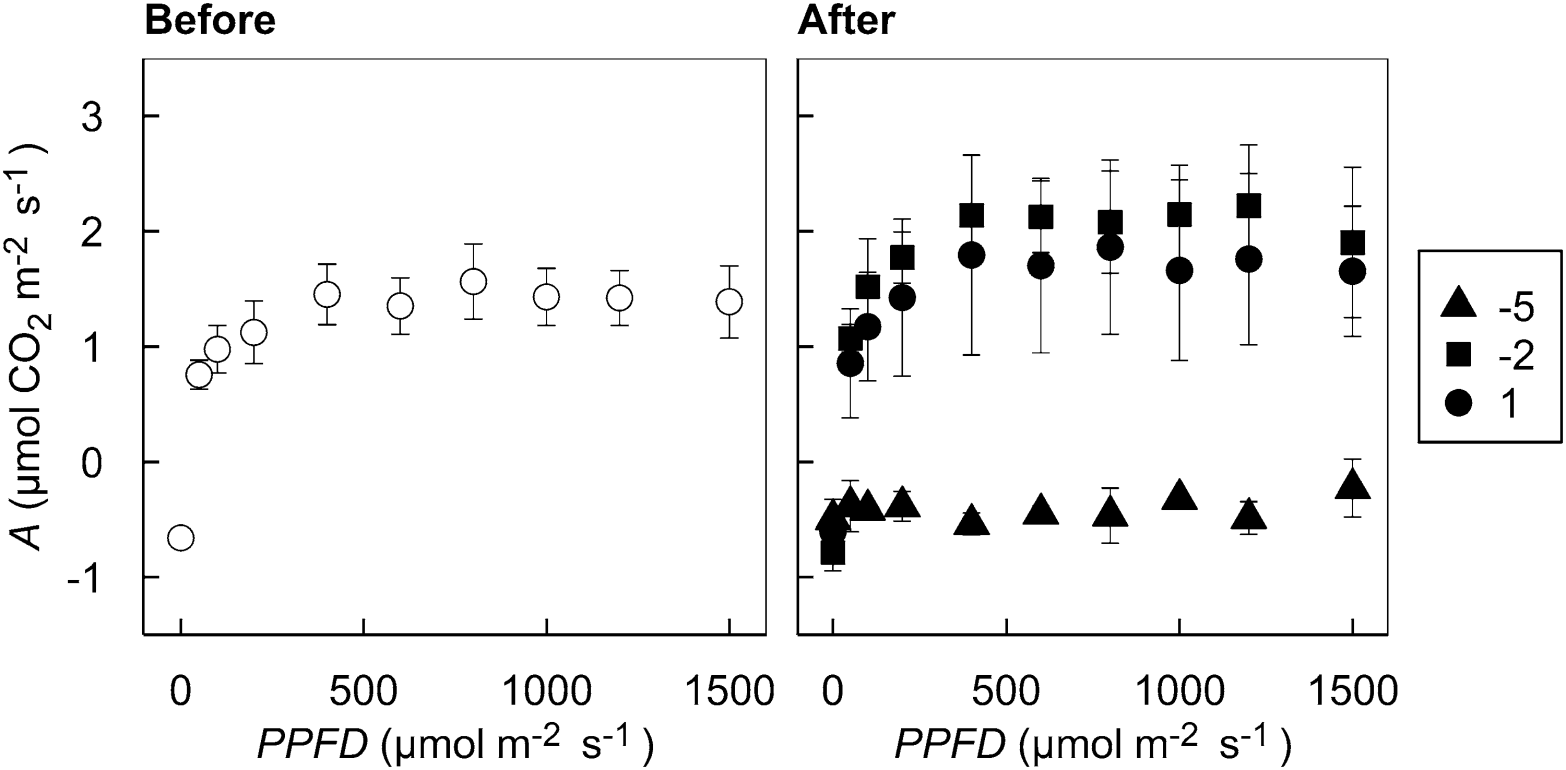
*A*-*PPFD* curves, net photosynthetic CO_2_ assimilation rate (*A*) as a function of photosynthetic photon flux density (*PPFD*) of three-year-old ‘Haas’ avocado seedling leaves before (left) and after (right) short-term low temperature treatments. All symbols present the mean responses of biological replications (*n* = 6 and 3 for before and after treatments, respectively). Error bars show the standard errors of the means for *A*. Closed triangles, squares, and circles after treatments indicate the responses of the samples after treated with 1 h 1, − 2, and − 5 °C, respectively.

Before short-term low temperature treatments, the ‘Hass’ avocado leaves showed signature responses of CO_2_ response curves (i.e., *A*–*C*_i_ curves; *A*, net CO_2_ assimilation rate as a function of *C*_i_, intercellular CO_2_ concentration of leaves) in C_3_ plants having both RuBisCo-limited photosynthesis up to approximately from 0–500 µmol CO_2_ mol^−1^ air and RuBP regeneration-limited photosynthesis from 500 µmol CO_2_ mol^−1^ air and above (Fig. 4)^43^. Triose-phosphate utilization-limited photosynthesis, however, was not observed in the samples, which may or may not be observed in C_3_ plants^44^. The maximum level of *A* was around 5 μmol CO_2_ m^−2^ s^−1^ observed at 1,100 µmol CO_2_ mol^−1^ air. Avocado trees have relatively low photosynthesis compared with other C_3_ fruit trees, showing various ranges of maximum photosynthetic rates under different growing conditions^45^. The actual *A* values under the ambient growing conditions at 400 µmol CO_2_ m^−2^ s^−1^ with saturating light intensity of 1500 *PPFD* were lower than those reported in the literature, measured 1.6 µmol CO_2_ m^−2^ s^−1^ before treatment (Figs. 4 and 5; Table 2). The *A* values of container-grown avocado were reported to be around 6 µmol CO_2_ m^−2^ s^−1^ in three-month-old avocado seedlings^46^ and 5.9–7.6 µmol CO_2_ m^−2^ s^−1^ in less than one-year-old grafted avocado seedlings^3^. As avocado plants grow *A* also increases, so it could reach up to 11 µmol CO_2_ m^−2^ s^−1^ when 18 months old^5^. A small container size of our experiment and a relatively low temperature during the growing period may have limited overall growth of the plants, represented by the decreased *A*.

**Table 2:** Descriptive and inferential statistics of photosynthetic parameters at the operational points of the CO_2_ (Fig. 2) and the light (Fig. 3) response curves.

Treatment	*A*^1^	*E*	*C*_i_	*g*_s_	*Φ*_PSII_	*Φ*_CO2_	Fv/Fm	qP	qN
	μmol CO_2_ m^−2^ s^−1^	mmol H_2_O m^−2^ s^−1^	µmol CO_2_ mol^−1^	mol H_2_O m^−2^ s^−1^	Unitless	Unitless	Unitless	Unitless	Unitless
Before	1.659 ± 0.258^2^a^3^	0.475 ± 0.066a	280.7 ± 14.1b	0.027 ± 0.005a	0.011 ± 0.001ab	0.0017 ± 0.0002a	0.625 ± 0.018a	0.029 ± 0.004b	0.641 ± 0.038b
1 °C	1.952 ± 0.528a	0.397 ± 0.085a	270.1 ± 33.6b	0.029 ± 0.007a	0.020 ± 0.005ab	0.0019 ± 0.0004a	0.684 ± 0.019a	0.047 ± 0.001ab	0.662 ± 0.053ab
–2 °C	2.035 ± 0.343a	0.356 ± 0.071ab	272.8 ± 29.0b	0.031 ± 0.007a	0.022 ± 0.004a	0.0020 ± 0.0003a	0.706 ± 0.017a	0.044 ± 0.007ab	0.584 ± 0.039b
–5 °C	–0.110 ± 0.125b	0.081 ± 0.018b	433.0 ± 31.6a	0.005 ± 0.001b	0.008 ± 0.003b	0.0002 ± 0.0001b	0.332 ± 0.083b	0.081 ± 0.028a	0.809 ± 0.035a
ANOVA	7.647 (0.000)***^4^	5.828 (0.003)**	8.095 (0.000)***	4.042 (0.017)*	4.593 (0.010)***	7.821 (0.000)***	17.56 (0.000)***	3.078 (0.045)*	4.184 (0.015)*

The means of biological replications are provided with the standard errors of the means in parentheses (n = 6). F-statistics of one-way ANOVA test results for low-temperature effect (df = 2) on these photosynthetic parameters are presented with statistical significance codes. The corresponding *p*-values of the F-statistics are presented in parentheses.

^1^*A*, the net CO_2_ assimilation rate; *E*, transpiration rate; *C*_i_, the intercellular CO_2_ concentration; *g*_s_, stomatal conductance to H_2_O; *Φ*_PSII_, the quantum yield of photosystem II estimated by fluorescence measurement; *Φ*_CO2_, the quantum yield of CO_2_ assimilation estimated by gas exchange measurement; Fv/Fm, the maximum quantum efficiency of photosystem II reaction centers; qP, photochemical quenching; qN, non-photochemical quenching.

^2^Means with ± 1 standard errors of the means from six replicates—three from *A*–*C*_i_ and *A*-*PPFD* curves each under the ambient 400 ppm CO_2_ and saturating light 1500 PPFD conditions.

^3^Means within columns followed by different lowercase letters are significantly different according to Tukey’s honestly significant difference test at *p* < 0.05.

^4^Statistical significance codes: *, **, and *** for *p* < 0.05, 0.001, and 0.001 levels, significantly.

After short-term low temperature treatments, the avocado leaves showed distinguished down-regulation of photosynthetic activity in the seedlings treated with − 5 °C while those in the seedlings treated with the other low temperatures did not inhibit the photosynthetic responses. At low levels of *C*_i_, less than 400 µmol CO_2_ mol^−1^ air, net CO_2_ assimilation turned to negative values, meaning that the leaves rather were respiring over the low *C*_i_ range. When subtropical or tropical plants were exposed to low temperatures, 0–12 °C can cause chilling injuries, while < 0 °C can cause more acute frost injuries, represented by the inhibition of photosynthesis from the ROS production^3^. In ‘Hass’ avocado, it was shown that an overnight chilling temperature reduced *A* about 65% on a subsequent day after the cold night^3^.

The avocado seedlings showed a characteristic of shade leaves, *A* being saturated at 500 µmol m^−2^ s^−1^ photosynthetic photon flux density (*PPFD*) as reported in Mandemaker^45^ (Fig. 5). The maximum level of *A* under saturating light intensity was around 2 µmol m^−2^ s^−1^ before treatment, which is lower than the previously reported values^3^. After short-term low temperature treatments, as shown in the *A*−*C*_i_ responses (Fig. 4), the light response curves (i.e., *A*-*PPFD* curve) presented an apparent down-regulated photosynthetic capacity in the seedlings treated with − 5 °C under saturating light conditions. Over all the *PPFD* measured, *A* remained negative, indicating the leaves were damaged after the − 5 °C treatment were respiring at all light levels. However, similarly to the *A*–*C*_i_ responses, the 1 and − 2 °C treatments both did not produce a significant reduction in *A*, although these are damaging low temperatures in avocado demonstrated in the literature^3^. This is likely because the plants used in our study were adapted to low temperatures for a few months even before treatment (average growing temperature of 10.53 °C and 11.06 °C for the two months prior to the experiment).

Under the ambient growing conditions at 400 µmol CO_2_ m^−2^ s^−1^ with saturating light intensity of 1500 *PPFD*, the avocado leaves in the seedlings treated with − 5 °C showed lower gas exchange and chlorophyll fluorescence values in *A*, *E*, *g*_s_, *Φ*_PSII_, *Φ*_CO2_, and Fv/Fm and higher values in *C*_i_, qP, and qN in comparison with the leaves in the seedlings before treatment and treated with 1 and − 2 °C (Table 2). The changes in these values indicate not only reduced photosynthetic CO_2_ assimilation and transpirational H_2_O release but also increased photoprotection operating to compensate for the decreased utilization of absorbed light energy harvested in PSII by chilling stress from low temperature. A notable decrease in *g*_s_ of the leaves in the seedlings treated with − 5 °C confirms the stomatal limitation of photosynthetic CO_2_ uptake. The subsequent negative *A* value along with increased *C*_i_ in the leaves in the seedlings treated with − 5 °C implies the damaged tissues by chilling stress only afforded respiration, not photosynthesis. Also, the non-stomatal limitation was observed in the following manner. Increased qP in the leaves in the seedlings treated with − 5 °C could be likely from increased photochemical quenching of absorbed light energy through photorespiration, not through photosynthesis. Such down-regulation, in turn, appeared to induce non-photochemical quenching of the excess energy, increased qN in the leaves in the seedlings treated with − 5 °C. These photosynthetic alterations were reported in the literature as adaptive responses to low temperature^47,48^.

Overall, from the response curve analysis, it was observed that the exposure to short-term low temperature led to stomatal and non-stomatal limitation of photosynthesis in avocado leaves, which only occurred at the leaves in the seedlings treated with − 5 °C, not at 1 and − 2 °C. In response to the downregulation of photosynthesis, the photoprotection in the leaves in the seedlings treated with − 5 °C was induced; nevertheless, the damaged PSII could not withstand saturating light intensity.

### Antioxidant activities

**Figure 6:**
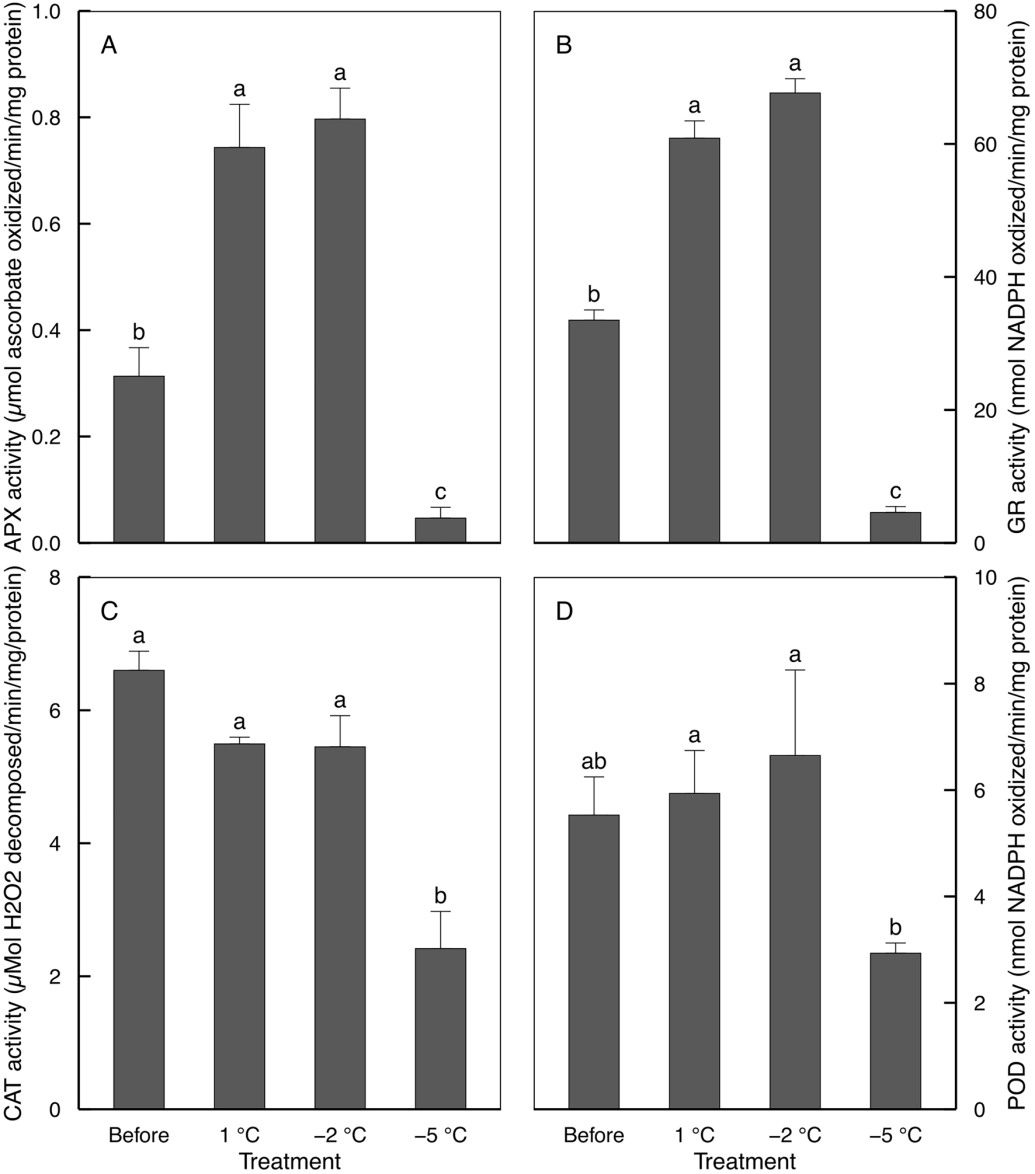
Antioxidative enzymes in three-year-old ‘Hass’ avocado seedling leaves before and after 24 h after exposure to 1, − 2, and − 5 °C for 1 h. Means with standard errors from three replicates with five leaf each. Means with bars followed by different lowercase letters are significantly different according to Tukey’s honestly significant difference test at *p* < 0.05.

In ‘Hass’ avocado seedlings, enzymatic antioxidant activities were assayed to examine whether individual antioxidant enzymes contribute to the stabilization of photosystems against oxidative stress by short-term low temperatures (Fig. 6). Since the activity of individual enzymes varied depending on plant types and growth conditions^17,40,42^, the activity would be too complicated to understand. Still, the relationship with photosystem in this study could be described as relatively more straightforward than other biochemical systems. Generally, an increase in the activities prevented the accumulation of H_2_O_2_ and consequent cellular damage, suggesting that enzymes may play an important role in plant responses to oxidative stress caused by low temperature^40^. In the leaves in the avocado seedlings treated with 1 and − 2 °C, APX and GR activities increased compared to those before treatment (Fig. 6A,B), while CAT and POD activities did not differ (Fig. 6C,D). This result suggests that ascorbate–glutathione pathway^49^ using APX and GR was stimulated to protect photosystem from oxidative stress in the leaves in the seedlings treated with 1 and − 2 °C. Especially, APX is known to be allocated to chloroplasts, cytosol, and even in peroxisomes^50^. Chloroplast APX existing as both soluble and thylakoid-bound forms provided effective elimination of H_2_O_2_ generated both internally and externally^50^. Although CAT and POD had antioxidant activity in response to chilling stress in other avocado cultivars^40^, the enzymes in ‘Hass’ avocado seedlings were not differently activated (Fig. 6C,D).

A decrease in the enzyme activities occur when enzymes were consumed to counteract the increased in ROS production in response to stress or were inactivated due to severe cell damage^40^. In the leaves in avocado seedlings treated with − 5 °C, the activities of APX, GR, and CAT decreased compared to those before treatment and treated with 1 and − 2 °C (Fig. 6A–C). POD activities significantly decreased compared to those not before treatment but treated with 1 and − 2 °C (Fig. 6D). A decrease in the enzymatic activities had been shown in plants, including avocados, when it was exposed to close to the lethal temperature^40^. Nevertheless, we need further study to identify the sequence of decreases in the activities of antioxidant enzymes in the leaves in the seedlings treated with − 5 °C.

**Figure 7:**
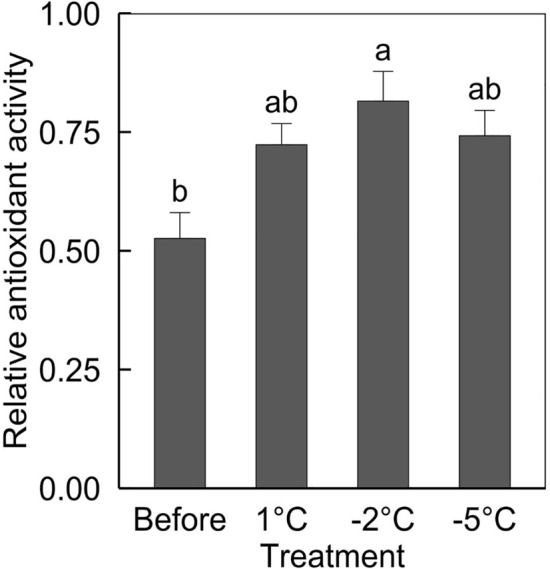
ABTS scavenging activity of three-year-old ‘Hass’ avocado seedling leaves before and after 24-h after exposure to 1, − 2, and − 5 °C for 1 h. Means with standard errors from three replicates with five leaf each. Means with bars followed by different lowercase letters are significantly different according to Tukey’s honestly significant difference test at *p* < 0.05.

In addition to the stimulation of enzymatic antioxidants, non-enzymatic antioxidants could be accumulated to scavenge ROS generated by chilling stress (Fig. 7)^19^. Compared to avocado seedling leaves before treatment, non-enzymatic antioxidant activity did not change in those treated with 1 and − 5 °C but increased significantly in those treated with − 2 °C. Although the contents of carotenoid and phenolics were the highest in those treated with − 5 °C than the others (Table 1), the antioxidant activity could not be affected by the accumulation of the compounds. Weil et al.^51^ reported, in ‘Hass’ avocado cultivar, a similar result with this study; the non-enzymatic antioxidant activity did not change under gradient decreased temperatures from 13 to − 2.9 °C with the duration of 2 h per each step. Meanwhile, the antioxidant activity of ‘Ettinger’ avocado cultivar, the greater cold tolerance than that of ‘Hass’, was greater than before treatment when exposed to the gradient temperature. In various plants, cold-tolerant cultivars have higher or an increase in antioxidant activity under low temperatures than cold-sensitive cultivars^51^. In this result, we confirmed ‘Hass’ avocado characteristics as a cold-sensitive cultivar; the non-enzymatic antioxidant activity was not stimulated regardless of the level of chilling stress, even under critical temperature, which caused the severe damage of photosystem.

## Conclusions

We investigated the effects of short-term low temperatures on physiological and biochemical responses in ‘Hass’ avocado cultivar for the cultivation in Republic of Korea, one of the new cultivation areas in temperate regions, where avocado has been just introduced. After being treated with 1 or − 2 °C for 1 h, the photosynthetic characteristics of the avocado leaves did not change compared to the leaves before treatment. Although oxidative stress occurred, the photosystem was protected by antioxidant enzymes, including APX and GR. After being treated with − 5 °C for 1 h, we identified the changes in morphology and pigment contents as a signal of physiological disorders. The avocado leaves showed remarkable reductions in photosynthetic activities; stomatal and non-stomatal limitations on photosynthesis lowered the capacity to process the light quanta. Under low-temperature conditions at − 5 °C, the excess light energy damaged PSII of the leaves with inactivated antioxidative mechanisms. These results indicated avocado seedlings suffer irreversible damage when exposed to the threshold level of low temperature, even for a short period of time. Therefore, ‘Hass’ avocado seedlings would be cultivated at least above − 5 °C, being a lethal temperature in the new cultivation regions of Republic of Korea.

## Data Availability

All data generated or analyzed during this study are included in this published article in form of figures and tables.
